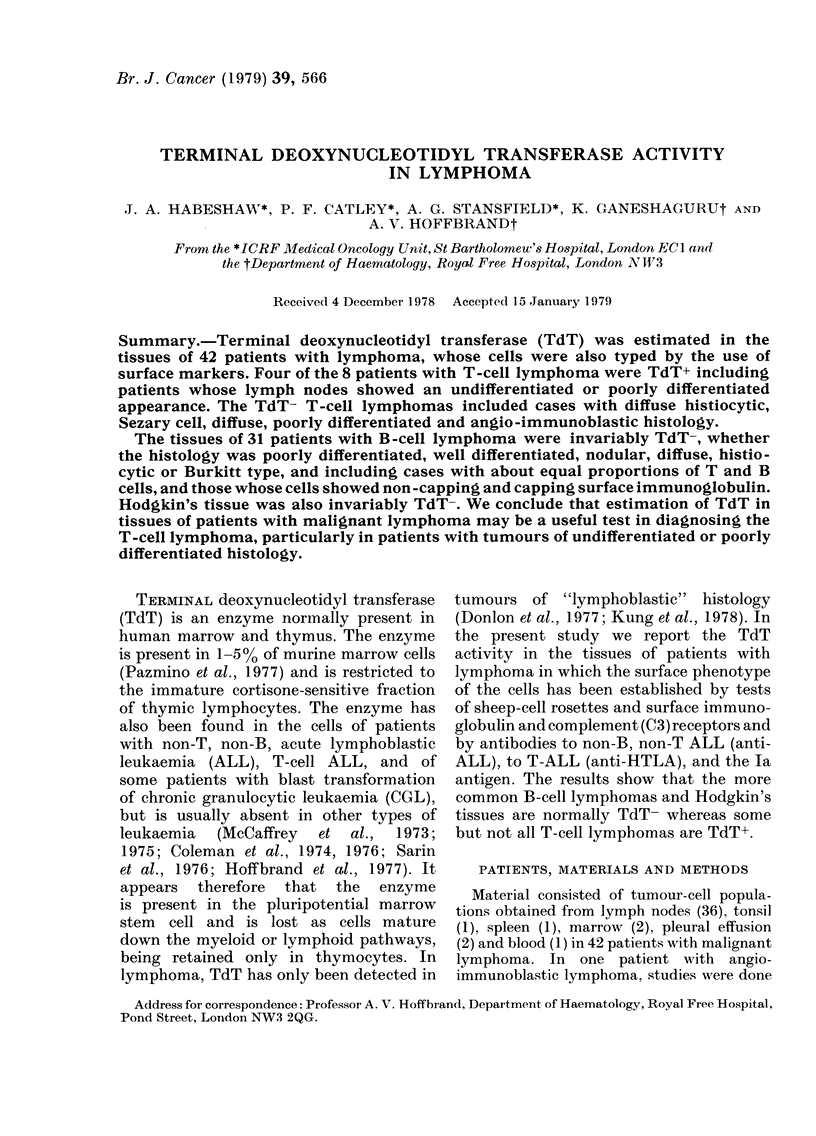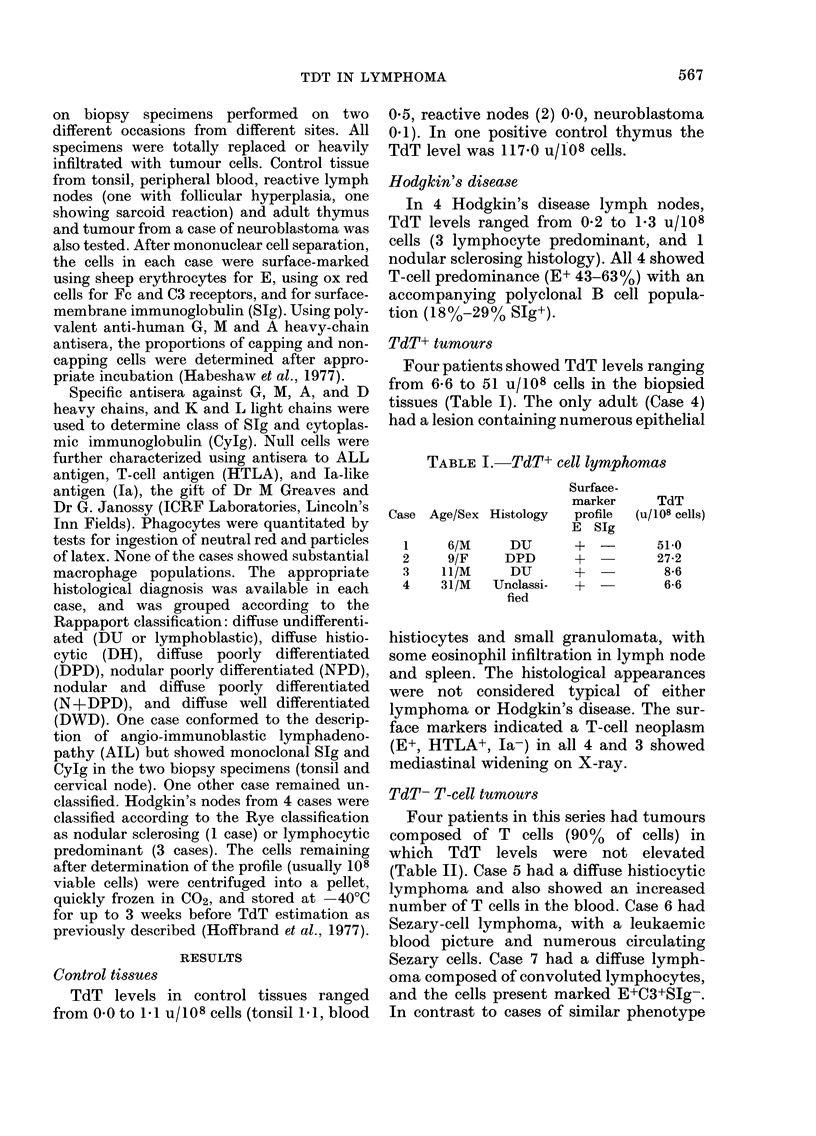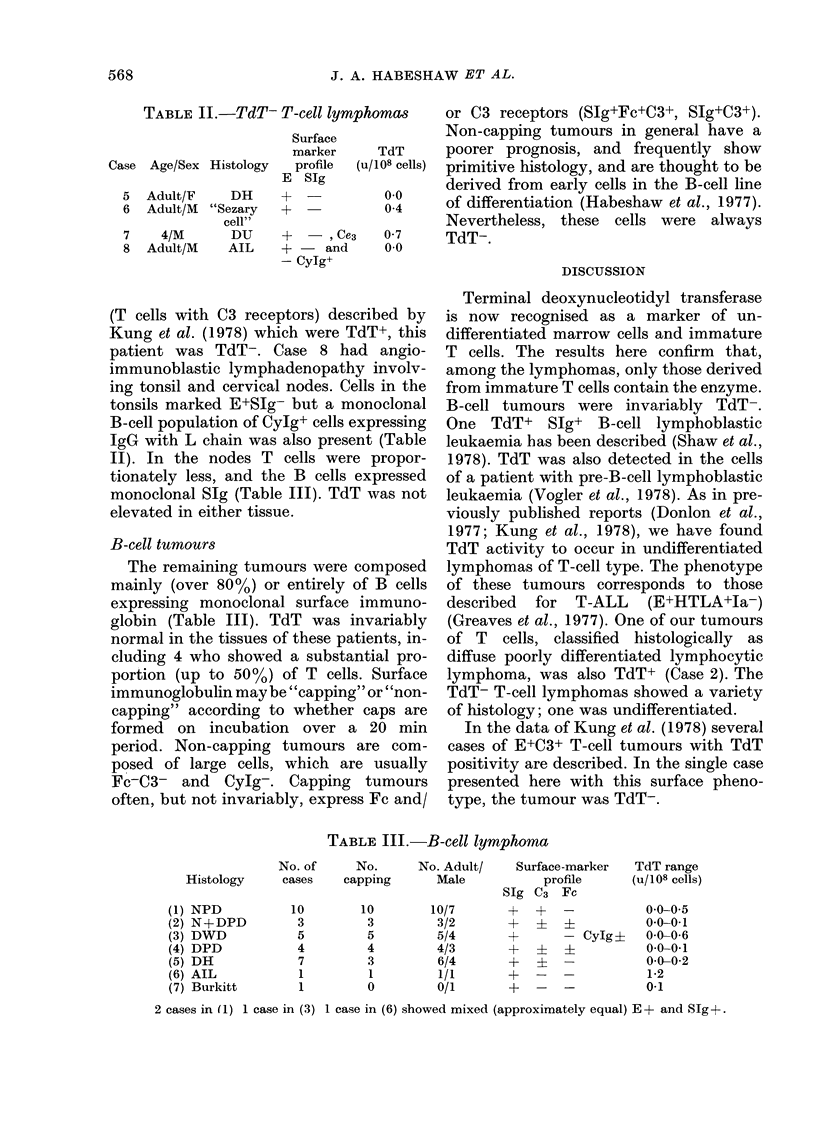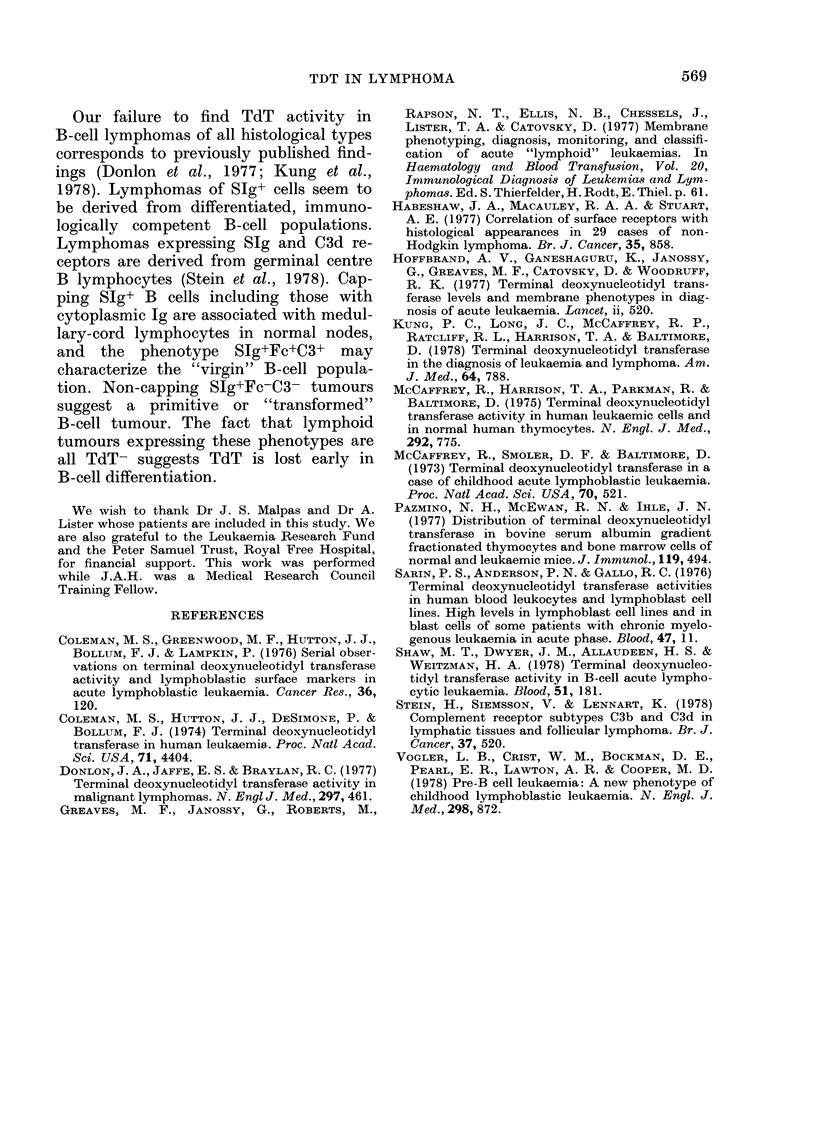# Terminal deoxynucleotidyl transferase activity in lymphoma.

**DOI:** 10.1038/bjc.1979.102

**Published:** 1979-05

**Authors:** J. A. Habeshaw, P. F. Catley, A. G. Stansfield, K. Ganeshaguru, A. V. Hoffbrand

## Abstract

Terminal deoxynucleotidyl transferase (TdT) was estimated in the tissues of 42 patients with lymphoma, whose cells were also typed by the use of surface markers. Four of the 8 patients with T-cell lymphoma were TdT+ including patients whose lymph nodes showed an undifferentiated or poorly differentiated appearance. The TdT- T-cell lymphomas included cases with diffuse histiocytic Sezary cell, diffuse, poorly differentiated and angio-immunoblastic histology. The tissues of 31 patients with B-cell lymphoma were invariably TdT-, whether the histology was poorly differentiated, well differentiated, nodular, diffuse, histiocytic or Burkitt type, and including cases with about equal proportions of T and B cells, and those whose cells showed non-capping and capping surface immunoglobulin. Hodgkin's tissue was also invariably TdT-. We conclude that estimation of TdT in tissues of patients with malignant lymphoma may be a useful test in diagnosing the T-cell lymphoma, particularly in patients with tumours of undifferentiated or poorly differentiated histology.


*Br. J. Cancer* (1979) **39**, 566

### TERMINAL DEOXYNUCLEOTIDYL TRANSFERASE ACTIVITY IN LYMPHOMA

J. A. HABESHAW*, P. F. CATLEY*, A. G. STANSFIELD*, K. GANESHAGURU† AND A. V. HOFFBRAND†

*From the *ICRF Medical Oncology Unit, St Bartholomew's Hospital, London EC1 and the †Department of Haematology, Royal Free Hospital, London NW3*

Received 4 December 1978 Accepted 15 January 1979

**Summary.—Terminal deoxynucleotidyl transferase (TdT) was estimated in the tissues of 42 patients with lymphoma, whose cells were also typed by the use of surface markers. Four of the 8 patients with T-cell lymphoma were TdT$^+$ including patients whose lymph nodes showed an undifferentiated or poorly differentiated appearance. The TdT$^-$ T-cell lymphomas included cases with diffuse histiocytic, Sezary cell, diffuse, poorly differentiated and angio-immunoblastic histology.**

**The tissues of 31 patients with B-cell lymphoma were invariably TdT$^-$, whether the histology was poorly differentiated, well differentiated, nodular, diffuse, histiocytic or Burkitt type, and including cases with about equal proportions of T and B cells, and those whose cells showed non-capping and capping surface immunoglobulin. Hodgkin's tissue was also invariably TdT$^-$. We conclude that estimation of TdT in tissues of patients with malignant lymphoma may be a useful test in diagnosing the T-cell lymphoma, particularly in patients with tumours of undifferentiated or poorly differentiated histology.**

TERMINAL deoxynucleotidyl transferase (TdT) is an enzyme normally present in human marrow and thymus. The enzyme is present in 1–5% of murine marrow cells (Pazmino *et al.*, 1977) and is restricted to the immature cortisone-sensitive fraction of thymic lymphocytes. The enzyme has also been found in the cells of patients with non-T, non-B, acute lymphoblastic leukaemia (ALL), T-cell ALL, and of some patients with blast transformation of chronic granulocytic leukaemia (CGL), but is usually absent in other types of leukaemia (McCaffrey *et al.*, 1973; 1975; Coleman *et al.*, 1974, 1976; Sarin *et al.*, 1976; Hoffbrand *et al.*, 1977). It appears therefore that the enzyme is present in the pluripotential marrow stem cell and is lost as cells mature down the myeloid or lymphoid pathways, being retained only in thymocytes. In lymphoma, TdT has only been detected in tumours of "lymphoblastic" histology (Donlon *et al.*, 1977; Kung *et al.*, 1978). In the present study we report the TdT activity in the tissues of patients with lymphoma in which the surface phenotype of the cells has been established by tests of sheep-cell rosettes and surface immunoglobulin and complement (C3) receptors and by antibodies to non-B, non-T ALL (anti-ALL), to T-ALL (anti-HTLA), and the Ia antigen. The results show that the more common B-cell lymphomas and Hodgkin's tissues are normally TdT$^-$ whereas some but not all T-cell lymphomas are TdT$^+$.

### PATIENTS, MATERIALS AND METHODS

Material consisted of tumour-cell populations obtained from lymph nodes (36), tonsil (1), spleen (1), marrow (2), pleural effusion (2) and blood (1) in 42 patients with malignant lymphoma. In one patient with angio-immunoblastic lymphoma, studies were done

Address for correspondence: Professor A. V. Hoffbrand, Department of Haematology, Royal Free Hospital, Pond Street, London NW3 2QG.

on biopsy specimens performed on two different occasions from different sites. All specimens were totally replaced or heavily infiltrated with tumour cells. Control tissue from tonsil, peripheral blood, reactive lymph nodes (one with follicular hyperplasia, one showing sarcoid reaction) and adult thymus and tumour from a case of neuroblastoma was also tested. After mononuclear cell separation, the cells in each case were surface-marked using sheep erythrocytes for E, using ox red cells for Fc and C3 receptors, and for surface-membrane immunoglobulin (SIg). Using polyvalent anti-human G, M and A heavy-chain antisera, the proportions of capping and non-capping cells were determined after appropriate incubation (Habeshaw *et al.*, 1977).

Specific antisera against G, M, A, and D heavy chains, and K and L light chains were used to determine class of SIg and cytoplasmic immunoglobulin (CyIg). Null cells were further characterized using antisera to ALL antigen, T-cell antigen (HTLA), and Ia-like antigen (Ia), the gift of Dr M Greaves and Dr G. Janossy (ICRF Laboratories, Lincoln's Inn Fields). Phagocytes were quantitated by tests for ingestion of neutral red and particles of latex. None of the cases showed substantial macrophage populations. The appropriate histological diagnosis was available in each case, and was grouped according to the Rappaport classification: diffuse undifferentiated (DU or lymphoblastic), diffuse histiocytic (DH), diffuse poorly differentiated (DPD), nodular poorly differentiated (NPD), nodular and diffuse poorly differentiated (N+DPD), and diffuse well differentiated (DWD). One case conformed to the description of angio-immunoblastic lymphadenopathy (AIL) but showed monoclonal SIg and CyIg in the two biopsy specimens (tonsil and cervical node). One other case remained unclassified. Hodgkin's nodes from 4 cases were classified according to the Rye classification as nodular sclerosing (1 case) or lymphocytic predominant (3 cases). The cells remaining after determination of the profile (usually $10^8$ viable cells) were centrifuged into a pellet, quickly frozen in $CO_2$, and stored at −40°C for up to 3 weeks before TdT estimation as previously described (Hoffbrand *et al.*, 1977).

### RESULTS

### *Control tissues*

TdT levels in control tissues ranged from 0·0 to 1·1 u/$10^8$ cells (tonsil 1·1, blood 0·5, reactive nodes (2) 0·0, neuroblastoma 0·1). In one positive control thymus the TdT level was 117·0 u/$10^8$ cells.

### *Hodgkin's disease*

In 4 Hodgkin's disease lymph nodes, TdT levels ranged from 0·2 to 1·3 u/$10^8$ cells (3 lymphocyte predominant, and 1 nodular sclerosing histology). All 4 showed T-cell predominance ($E^+$ 43–63%) with an accompanying polyclonal B cell population (18%–29% $SIg^+$).

### *$TdT^+$ tumours*

Four patients showed TdT levels ranging from 6·6 to 51 u/$10^8$ cells in the biopsied tissues (Table I). The only adult (Case 4) had a lesion containing numerous epithelial histiocytes and small granulomata, with some eosinophil infiltration in lymph node and spleen. The histological appearances were not considered typical of either lymphoma or Hodgkin's disease. The surface markers indicated a T-cell neoplasm ($E^+$, $HTLA^+$, $Ia^-$) in all 4 and 3 showed mediastinal widening on X-ray.

TABLE I.—*$TdT^+$ cell lymphomas*

| Case | Age/Sex | Histology | Surface-marker profile E | SIg | TdT (u/$10^8$ cells) |
|---|---|---|---|---|---|
| 1 | 6/M | DU | + | — | 51·0 |
| 2 | 9/F | DPD | + | — | 27·2 |
| 3 | 11/M | DU | + | — | 8·6 |
| 4 | 31/M | Unclassified | + | — | 6·6 |

### *$TdT^-$ T-cell tumours*

Four patients in this series had tumours composed of T cells (90% of cells) in which TdT levels were not elevated (Table II). Case 5 had a diffuse histiocytic lymphoma and also showed an increased number of T cells in the blood. Case 6 had Sezary-cell lymphoma, with a leukaemic blood picture and numerous circulating Sezary cells. Case 7 had a diffuse lymphoma composed of convoluted lymphocytes, and the cells present marked $E^+C3^+SIg^-$. In contrast to cases of similar phenotype

TABLE II.—*TdT⁻ T-cell lymphomas*

| Case | Age/Sex | Histology | Surface marker profile E | SIg | TdT (u/$10^8$ cells) |
|---|---|---|---|---|---|
| 5 | Adult/F | DH | + | — | 0·0 |
| 6 | Adult/M | "Sezary cell" | + | — | 0·4 |
| 7 | 4/M | DU | + | — , $Ce_3$ | 0·7 |
| 8 | Adult/M | AIL | + | — and – $CyIg^+$ | 0·0 |

(T cells with C3 receptors) described by Kung *et al.* (1978) which were $TdT^+$, this patient was $TdT^-$. Case 8 had angio-immunoblastic lymphadenopathy involving tonsil and cervical nodes. Cells in the tonsils marked $E^+SIg^-$ but a monoclonal B-cell population of $CyIg^+$ cells expressing IgG with L chain was also present (Table II). In the nodes T cells were proportionately less, and the B cells expressed monoclonal SIg (Table III). TdT was not elevated in either tissue.

*B-cell tumours*

The remaining tumours were composed mainly (over 80%) or entirely of B cells expressing monoclonal surface immunoglobin (Table III). TdT was invariably normal in the tissues of these patients, including 4 who showed a substantial proportion (up to 50%) of T cells. Surface immunoglobulin may be "capping" or "non-capping" according to whether caps are formed on incubation over a 20 min period. Non-capping tumours are composed of large cells, which are usually $Fc^-C3^-$ and $CyIg^-$. Capping tumours often, but not invariably, express Fc and/or C3 receptors ($SIg^+Fc^+C3^+$, $SIg^+C3^+$). Non-capping tumours in general have a poorer prognosis, and frequently show primitive histology, and are thought to be derived from early cells in the B-cell line of differentiation (Habeshaw *et al.*, 1977). Nevertheless, these cells were always $TdT^-$.

### DISCUSSION

Terminal deoxynucleotidyl transferase is now recognised as a marker of undifferentiated marrow cells and immature T cells. The results here confirm that, among the lymphomas, only those derived from immature T cells contain the enzyme. B-cell tumours were invariably $TdT^-$. One $TdT^+$ $SIg^+$ B-cell lymphoblastic leukaemia has been described (Shaw *et al.*, 1978). TdT was also detected in the cells of a patient with pre-B-cell lymphoblastic leukaemia (Vogler *et al.*, 1978). As in previously published reports (Donlon *et al.*, 1977; Kung *et al.*, 1978), we have found TdT activity to occur in undifferentiated lymphomas of T-cell type. The phenotype of these tumours corresponds to those described for T-ALL ($E^+HTLA^+Ia^-$) (Greaves *et al.*, 1977). One of our tumours of T cells, classified histologically as diffuse poorly differentiated lymphocytic lymphoma, was also $TdT^+$ (Case 2). The $TdT^-$ T-cell lymphomas showed a variety of histology; one was undifferentiated.

In the data of Kung *et al.* (1978) several cases of $E^+C3^+$ T-cell tumours with TdT positivity are described. In the single case presented here with this surface phenotype, the tumour was $TdT^-$.

TABLE III.—*B-cell lymphoma*

| Histology | No. of cases | No. capping | No. Adult/ Male | Surface-marker profile SIg | $C_3$ | Fc | | TdT range (u/$10^8$ cells) |
|---|---|---|---|---|---|---|---|---|
| (1) NPD | 10 | 10 | 10/7 | + | + | – | | 0·0–0·5 |
| (2) N+DPD | 3 | 3 | 3/2 | + | ± | ± | | 0·0–0·1 |
| (3) DWD | 5 | 5 | 5/4 | + | | – | CyIg± | 0·0–0·6 |
| (4) DPD | 4 | 4 | 4/3 | + | ± | ± | | 0·0–0·1 |
| (5) DH | 7 | 3 | 6/4 | + | ± | – | | 0·0–0·2 |
| (6) AIL | 1 | 1 | 1/1 | + | – | – | | 1·2 |
| (7) Burkitt | 1 | 0 | 0/1 | + | – | – | | 0·1 |

2 cases in (1) 1 case in (3) 1 case in (6) showed mixed (approximately equal) E+ and SIg+.

Our failure to find TdT activity in B-cell lymphomas of all histological types corresponds to previously published findings (Donlon *et al.*, 1977; Kung *et al.*, 1978). Lymphomas of $SIg^+$ cells seem to be derived from differentiated, immunologically competent B-cell populations. Lymphomas expressing SIg and C3d receptors are derived from germinal centre B lymphocytes (Stein *et al.*, 1978). Capping $SIg^+$ B cells including those with cytoplasmic Ig are associated with medullary-cord lymphocytes in normal nodes, and the phenotype $SIg^+Fc^+C3^+$ may characterize the "virgin" B-cell population. Non-capping $SIg^+Fc^-C3^-$ tumours suggest a primitive or "transformed" B-cell tumour. The fact that lymphoid tumours expressing these phenotypes are all $TdT^-$ suggests TdT is lost early in B-cell differentiation.

We wish to thank Dr J. S. Malpas and Dr A. Lister whose patients are included in this study. We are also grateful to the Leukaemia Research Fund and the Peter Samuel Trust, Royal Free Hospital, for financial support. This work was performed while J.A.H. was a Medical Research Council Training Fellow.

### REFERENCES

Coleman, M. S., Greenwood, M. F., Hutton, J. J., Bollum, F. J. & Lampkin, P. (1976) Serial observations on terminal deoxynucleotidyl transferase activity and lymphoblastic surface markers in acute lymphoblastic leukaemia. *Cancer Res.*, **36**, 120.

Coleman, M. S., Hutton, J. J., DeSimone, P. & Bollum, F. J. (1974) Terminal deoxynucleotidyl transferase in human leukaemia. *Proc. Natl Acad. Sci. USA*, **71**, 4404.

Donlon, J. A., Jaffe, E. S. & Braylan, R. C. (1977) Terminal deoxynucleotidyl transferase activity in malignant lymphomas. *N. Engl J. Med.*, **297**, 461.

Greaves, M. F., Janossy, G., Roberts, M., Rapson, N. T., Ellis, N. B., Chessels, J., Lister, T. A. & Catovsky, D. (1977) Membrane phenotyping, diagnosis, monitoring, and classification of acute "lymphoid" leukaemias. In *Haematology and Blood Transfusion, Vol. 20, Immunological Diagnosis of Leukemias and Lymphomas.* Ed. S. Thierfelder, H. Rodt, E. Thiel. p. 61.

Habeshaw, J. A., Macauley, R. A. A. & Stuart, A. E. (1977) Correlation of surface receptors with histological appearances in 29 cases of non-Hodgkin lymphoma. *Br. J. Cancer*, **35**, 858.

Hoffbrand, A. V., Ganeshaguru, K., Janossy, G., Greaves, M. F., Catovsky, D. & Woodruff, R. K. (1977) Terminal deoxynucleotidyl transferase levels and membrane phenotypes in diagnosis of acute leukaemia. *Lancet*, ii, 520.

Kung, P. C., Long, J. C., McCaffrey, R. P., Ratcliff, R. L., Harrison, T. A. & Baltimore, D. (1978) Terminal deoxynucleotidyl transferase in the diagnosis of leukaemia and lymphoma. *Am. J. Med.*, **64**, 788.

McCaffrey, R., Harrison, T. A., Parkman, R. & Baltimore, D. (1975) Terminal deoxynucleotidyl transferase activity in human leukaemic cells and in normal human thymocytes. *N. Engl. J. Med.*, **292**, 775.

McCaffrey, R., Smoler, D. F. & Baltimore, D. (1973) Terminal deoxynucleotidyl transferase in a case of childhood acute lymphoblastic leukaemia. *Proc. Natl Acad. Sci. USA*, **70**, 521.

Pazmino, N. H., McEwan, R. N. & Ihle, J. N. (1977) Distribution of terminal deoxynucleotidyl transferase in bovine serum albumin gradient fractionated thymocytes and bone marrow cells of normal and leukaemic mice. *J. Immunol.*, **119**, 494.

Sarin, P. S., Anderson, P. N. & Gallo, R. C. (1976) Terminal deoxynucleotidyl transferase activities in human blood leukocytes and lymphoblast cell lines. High levels in lymphoblast cell lines and in blast cells of some patients with chronic myelogenous leukaemia in acute phase. *Blood*, **47**, 11.

Shaw, M. T., Dwyer, J. M., Allaudeen, H. S. & Weitzman, H. A. (1978) Terminal deoxynucleotidyl transferase activity in B-cell acute lymphocytic leukaemia. *Blood*, **51**, 181.

Stein, H., Siemsson, V. & Lennart, K. (1978) Complement receptor subtypes C3b and C3d in lymphatic tissues and follicular lymphoma. *Br. J. Cancer*, **37**, 520.

Vogler, L. B., Crist, W. M., Bockman, D. E., Pearl, E. R., Lawton, A. R. & Cooper, M. D. (1978) Pre-B cell leukaemia: A new phenotype of childhood lymphoblastic leukaemia. *N. Engl. J. Med.*, **298**, 872.